# Investigating ionizing radiation-induced changes in breast cancer cells using stimulated Raman scattering microscopy

**DOI:** 10.1117/1.JBO.28.7.076501

**Published:** 2023-07-11

**Authors:** Christian Harry Allen, Robyn Skillings, Duale Ahmed, Sarita Cuadros Sanchez, Kaitlyn Altwasser, George Hilan, William G. Willmore, Vinita Chauhan, Edana Cassol, Sangeeta Murugkar

**Affiliations:** a Carleton University, Department of Physics, Ottawa, Ontario, Canada; b Carleton University, Ottawa-Carleton Institute for Biomedical Engineering, Ottawa, Ontario, Canada; c Carleton University, Department of Health Sciences, Ottawa, Ontario, Canada; d Health Canada, Consumer and Clinical Radiation Protection Bureau, Ottawa, Ontario, Canada; e Carleton University, Institute of Biochemistry, Departments of Biology and Chemistry, Ottawa, Canada

**Keywords:** stimulated Raman scattering microscopy, ionizing radiation, lipids imaging

## Abstract

**Significance:**

Altered lipid metabolism of cancer cells has been implicated in increased radiation resistance. A better understanding of this phenomenon may lead to improved radiation treatment planning. Stimulated Raman scattering (SRS) microscopy enables label-free and quantitative imaging of cellular lipids but has never been applied in this domain.

**Aim:**

We sought to investigate the radiobiological response in human breast cancer MCF7 cells using SRS microscopy, focusing on how radiation affects lipid droplet (LD) distribution and cellular morphology.

**Approach:**

MCF7 breast cancer cells were exposed to either 0 or 30 Gy (X-ray) ionizing radiation and imaged using a spectrally focused SRS microscope every 24 hrs over a 72-hr time period. Images were analyzed to quantify changes in LD area per cell, lipid and protein content per cell, and cellular morphology. Cell viability and confluency were measured using a live cell imaging system while radiation-induced lipid peroxidation was assessed using BODIPY C11 staining and flow cytometry.

**Results:**

The LD area per cell and total lipid and protein intensities per cell were found to increase significantly for irradiated cells compared to control cells from 48 to 72 hrs post irradiation. Increased cell size, vacuole formation, and multinucleation were observed as well. No significant cell death was observed due to irradiation, but lipid peroxidation was found to be greater in the irradiated cells than control cells at 72 hrs.

**Conclusions:**

This pilot study demonstrates the potential of SRS imaging for investigating ionizing radiation-induced changes in cancer cells without the use of fluorescent labels.

## 1 Introduction

Radiation therapy (RT) is a standard treatment used in ∼50% of cancers worldwide. Its goal is to maximize tumor cell death while minimizing radiation-induced damage to healthy tissue.^1^ Despite the tremendous progress made in improving the efficacy of RT, radiation resistance in cancer cells and radiation toxicity in nearby healthy tissue are recognized as major problems in RT.^2^ The quest for solutions has led to improved technologies for delivering the RT dose,^3^ e.g., intensity-modulated RT (IMRT) and stereotactic ablative radiotherapy (SART). It has also led to increased efforts to gain a better understanding of the biological effects of radiation on cancer cells and the tumor microenvironment,^1^^–^^3^ with the aim of identifying the molecular mechanisms of radiation resistance and toxicity of bystander cells. Ionizing radiation causes direct damage to DNA, including single strand and double strand breaks. In addition, it induces water radiolysis, producing free radicals such as reactive oxygen species (ROS) that can cause lipid, protein, and DNA base modification.^1^^,^^4^^–^^6^ Increasing attention has been focused on the role of fatty acid metabolism in the radiation-induced response of cancer cells.^7^ Dysregulation in lipid metabolism of cancer cells^8^ has been implicated in increased radiation resistance^9^ in addition to cancer progression and aggressiveness.^10^ Hence quantitative methods to detect radiation-induced changes in lipid droplets (LDs) and lipid metabolism in cancer cells are required.

Previous studies have employed confocal fluorescence microscopy with lipid-conjugated or lipid-binding fluorophore probes to visualize changes in cellular LDs after radiation exposure of human cancer cells^9^ and murine cancer cells^11^ of distinct origins including from the lung, breast, bladder, and colon. The fluorophore probes, such as Oil Red O and Nile Red, have an associated risk of altering the inherent biophysical properties of the lipids thus affecting the native dynamics of LDs.^12^ Label-free imaging techniques based on Raman spectroscopy avoid such limitations. Raman spectroscopy involves the inelastic scattering of light due to vibrations of molecular bonds; it provides biochemical signatures of multiple macromolecules in a single acquisition. Over the past decade, Raman spectroscopy has been demonstrated as a formidable technique to investigate effects of ionizing radiation in cells and tissue.^13^^–^^15^ However, since the spontaneous Raman signal is very weak, long acquisition times are involved for imaging thus making it impractical for high resolution imaging of subcellular organelles including LDs. In fact, there are only a limited number of studies reporting the application of spontaneous Raman microscopy to assess radiation-induced changes to LDs in cancer cells.^16^^–^^18^ In contrast, coherent Raman imaging consisting of coherent anti-Stokes Raman scattering (CARS) and stimulated Raman scattering (SRS), relies on coherent Raman scattering and offers more than 1000-times faster imaging speed with subcellular resolution.^19^ SRS imaging greatly simplifies extraction of quantitative information and has significantly lower background compared to CARS imaging. SRS imaging of the significant high density of endogenous C-H chemical bonds (2800 to $3100\;{\mathrm{cm}}^{-1}$) has been demonstrated as an effective approach to image the biochemical response associated with cancer induced changes in DNA, lipids, and proteins of cells and tissue.^19^^–^^21^ In particular, SRS imaging was used to investigate the lipid metabolism^18^ in cell models of prostate cancer^10^ and ovarian cancer,^22^ as well as to probe drug-cell interactions in prostate^23^ and breast cancer cells.^24^ Coherent Raman imaging has never been applied to measure the radiobiological response of cancer cells, to the best of our knowledge.

Here we expand upon our preliminary findings^25^ and report the application of SRS imaging to detect the radiobiological response of human cancer cells for the first time. Human epithelial breast cancer (MCF7) cells were used as the model since their radiobiological response has been well characterized using confocal microscopy and Raman spectroscopy.^9^^,^^12^^,^^13^ The SRS microscopy images were acquired in the C-H stretching region (2800 to $3100\;{\mathrm{cm}}^{-1}$) from live MCF7 cells at three time points: 24, 48, and 72 hrs after exposure to 0 Gy (no irradiation) and 30 Gy of X-ray ionizing radiation. We characterized the radiation-induced effects on the LD area per cell, lipid and protein content per cell, as well as the cellular area and morphology as a function of dose and time. Cell viability and confluency were measured using a live cell imaging system, and radiation-induced lipid peroxidation was assessed using BODIPY C11 staining and flow cytometry. This work thus substantially builds upon our previous report,^25^ which only assessed the LD area per cell in the irradiated and control MCF7 cells at the different time points. Our results demonstrate that SRS imaging is a promising method for assessing the subcellular radiobiological response in MCF7 breast cancer cells.

## 2 Materials and Methods

### 2.1 SRS Imaging Setup

Our SRS imaging system consists of a dual output femtosecond (80 MHz repetition rate) laser (InSight DS+, MKS) with tunable (680 to 1300 nm) and fixed (1040 nm) outputs, a delay line to attain and adjust beam overlap, and a custom-built laser scanning microscope.^26^ Spectral focusing is achieved using compact adjustable-dispersion TIH53 glass blocks^27^ to chirp the femtosecond pulses into the picosecond domain, at 2.06 ps and 1.1 ps for pump and Stokes, respectively, and adjusting pulse overlap with the delay line, allowing for an $∼28\;{\mathrm{cm}}^{-1}$ spectral resolution. We measured the stimulated Raman loss (SRL) signal using lock-in detection with the Stokes beam modulated by an acousto-optic modulator (1205C-2, Isomet) at 4.9 MHz. The pump beam intensity was detected by a 1×1  cm photodiode (FDS1010, Thorlabs) biased to 60 V, filtered through a 2 to 7 MHz electronic bandpass filter, and delivered to a lock-in amplifier (MFLI, Zurich instruments) to extract the SRL signal. A pixel dwell time of 20  μs was used for the SRS image. ScanImage^28^ (Version 5.6, Vidrio Technologies) was used for control of the laser scanning and image acquisition.

### 2.2 Samples

Human epithelial breast tumor (MCF7) cells were cultured in Dulbecco’s Modified Eagle medium supplemented with 10% fetal bovine serum and 1% penicillin-streptomycin (PenStrep). Approximately $5\times 10^{5}$ MCF7 cells were seeded onto 35-mm round glass-bottom dishes (MatTek). Cells were allowed to incubate for 24 hrs post-seeding to achieve 50% confluency. The cells were irradiated using an X-ray irradiator (X-Rad320, Precision Xray) with 120 kVp X-rays and a $6.5^{\prime \prime }\times 6.5^{\prime \prime }$ irradiation field. Half of the cell culture dishes were irradiated at 30 Gy (1  Gy/minute) while the other half were left unirradiated as a control group. The cell culture dishes were left in the incubator for 24, 48, and 72 hrs post exposure.

Samples of dimethyl sulfoxide (DMSO) (2914 and $2998\;{\mathrm{cm}}^{-1}$) and polystyrene (2852 and $2904\;{\mathrm{cm}}^{-1}$) were used for calibrating the position of the delay line in terms of the Raman shifts in wavenumbers. Oleic acid and bovine serum albumin (BSA) were used as lipid and protein analogs for pure component estimates used in spectral unmixing of SRS images using multivariate curve resolution alternating least squares (MCR-ALS) as explained below.

### 2.3 Confluency and Cell Viability

To assess the effects of irradiation on cell confluency, $5\times 10^{5}$ MCF7 cells were seeded per well in tissue coated six-well plates to achieve 50% cell confluency 24 hrs post-seeding. After 24 hrs, cells were irradiated following the protocol described in Sec. 2.2. Control (unirradiated) and irradiated cells were imaged using the IncuCyte S3 Live Cell Imaging system (Sartorius). Images were taken in the phase contrast channel at 10× magnification every 4 hrs for a total of 96 hrs. Image analysis was performed using IncuCyte analysis software. Confluency was measured as the percentage (%) area covered by adherent cells in the culture dish. For cell viability assays, cells were stained with the viability dye 200 nM YOYO-1 (ThermoFisher) for 15 mins before imaging in the phase contrast and GFP channels. The % cell death was evaluated as the area of cells with the YOYO-1 fluorescence divided by the area of the MCF7 cells.

### 2.4 SRS Imaging of Cells

SRS spectra of DMSO (796 nm) and polystyrene (802 nm) were obtained each day to attain a linear map of Raman shift as a function of delay line position. The cells remained in the incubator until SRS imaging, with a pair of control (unirradiated, 0 Gy) sample and irradiated (30 Gy) sample being imaged on the same day. The growth medium was removed, and phosphate-buffered saline (PBS) was added to the cells just before SRS imaging. Approximately 10 regions in a single cell-culture dish were imaged using a 40× water immersion microscope objective (NA=0.8, Olympus) over an approximate 70 min period. SRS images of the cells were acquired in triplicate at $2850\;{\mathrm{cm}}^{-1}$ (802 nm pump), $2926\;{\mathrm{cm}}^{-1}$ (796 nm pump) for each time point. Beam powers at the sample were 30 mW for the pump beam and 25 mW for the Stokes; the laser power was set at a safe power to avoid damaging the cells, with combined peak intensities ($2.5\times 10^{10}\;W/{\mathrm{cm}}^{2}$) well below optical breakdown threshold ($2\times 10^{12}\;W/{\mathrm{cm}}^{2}$) and combined average power well below the recommended 100 mW limit for significant temperature changes.^29^^,^^30^ Images were obtained at 512×512  pixel resolution, with 20  μs pixel dwell-time, and averaged over 10 frames for each Raman band.

### 2.5 Lipid Peroxidation Assay

BODIPY (boron-dipyrromethene) 581/591 C11 (ThermoFisher Scientific) was used to measure lipid peroxidation through its shift of fluorescence emission measured at 490 to 510 nm upon interaction with peroxyl radicals. MCF7 cells ($5\times 10^{5}$ cells) were seeded onto 35 mm round poly-l-lysine-coated plates. Cells were allowed to incubate for 24 hrs post-seeding to achieve 50% confluency. Cells were then irradiated using the method described in Sec. 2.2. At the 24, 48, and 72-hr-time points post-irradiation, cells were washed three times with PBS, stained with BODIPY™ 581/591 C11 (2  μM final concentration) in PBS, and stored at 37°C in the dark for 10 min. For flow cytometry, cells were washed with PBS and harvested by trypsinization. Trypsinization was stopped with media and cells were centrifuged at 1,000 x g for 5 min. Media was removed and cells were resuspended in PBS and counted with an Attune NxT Flow Cytometer (ThermoFisher Scientific). Results were analyzed with FlowJo 10.4 software where the samples were gated to isolate single cells. The first plot (SSC: side scatter versus FSC: forward scatter) was gated (using polygon gating) to isolate cells that represented forward and side scattering indicative of live single and groups of cells based on their size and granularity. A second plot was generated from the first one that would gate (polygon gating) to further isolate the population of the chosen cells to only have single cells (SSC-H: side scatter high versus SSC-A: side scatter area). The single cell plots were used to create the log axis histograms in Fig. 6. All gates were consistently the same among every sample. The relative increase (%) in mean fluorescence intensity (MFI) due to peroxidation was calculated using the equation

$$\frac{\left|30\;\mathrm{Gy}\;\mathrm{MFI}-0\;\mathrm{Gy}\;\mathrm{MFI}\right|}{0\;\mathrm{Gy}\;\mathrm{MFI}}*100,$$ (1)

where 30 Gy MFI is the peroxidation induced MFI of the 30 Gy set, and 0 Gy MFI is the peroxidation induced MFI of the control set.

### 2.6 Image Processing and Statistical Analysis

Raw SRS images were averaged over the 10 frames taken at each Raman band, and the average images were then spectrally unmixed using MCR-ALS, in which images taken at different spectral bands are used, along with pure component intensities at those bands, to estimate images corresponding to pure components.^31^ Spectra from oleic acid and BSA were used as analogs for pure lipid and protein components. Their SRS spectra were min-max normalized and their intensities at 2850 and $2926\;{\mathrm{cm}}^{-1}$ used in the MCR-ALS spectral unmixing. Otsu thresholding was applied iteratively, with the criteria for the thresholding mask for LD segmentation determined from a comparison with the spectrally unmixed SRS images at 2850 and $2926\;{\mathrm{cm}}^{-1}$. LD area per cell was taken as the area of the thresholding mask (true pixels at $22.5\;\mu m^{2}$ per pixel) divided by the number of cells in a region. The average total lipid and protein intensities per cell were quantified by summing their respective intensities in each region and dividing by the cell count. Cellular area was evaluated by summing the protein area of the threshold mask of a given image and dividing by the number of cells. The LD area per cell, total lipid and protein intensities per cell, and cellular area values for irradiated and control sets were compared for each day using a two-sided Wilcoxon rank sum test. Equivalent to the two-sided Mann-Whitney U test, this is a nonparametric alternative to Student’s t-test, with an important difference being that the hypothesis being tested is whether samples from the two sets are from populations with equal medians, rather than equal means as in Student’s t-test. All image processing and analysis was done in MATLAB 2022b.

## 3 Results

### 3.1 Radiation-Induced Effects on Cell Confluency and Cell Viability

**Fig. 1:**
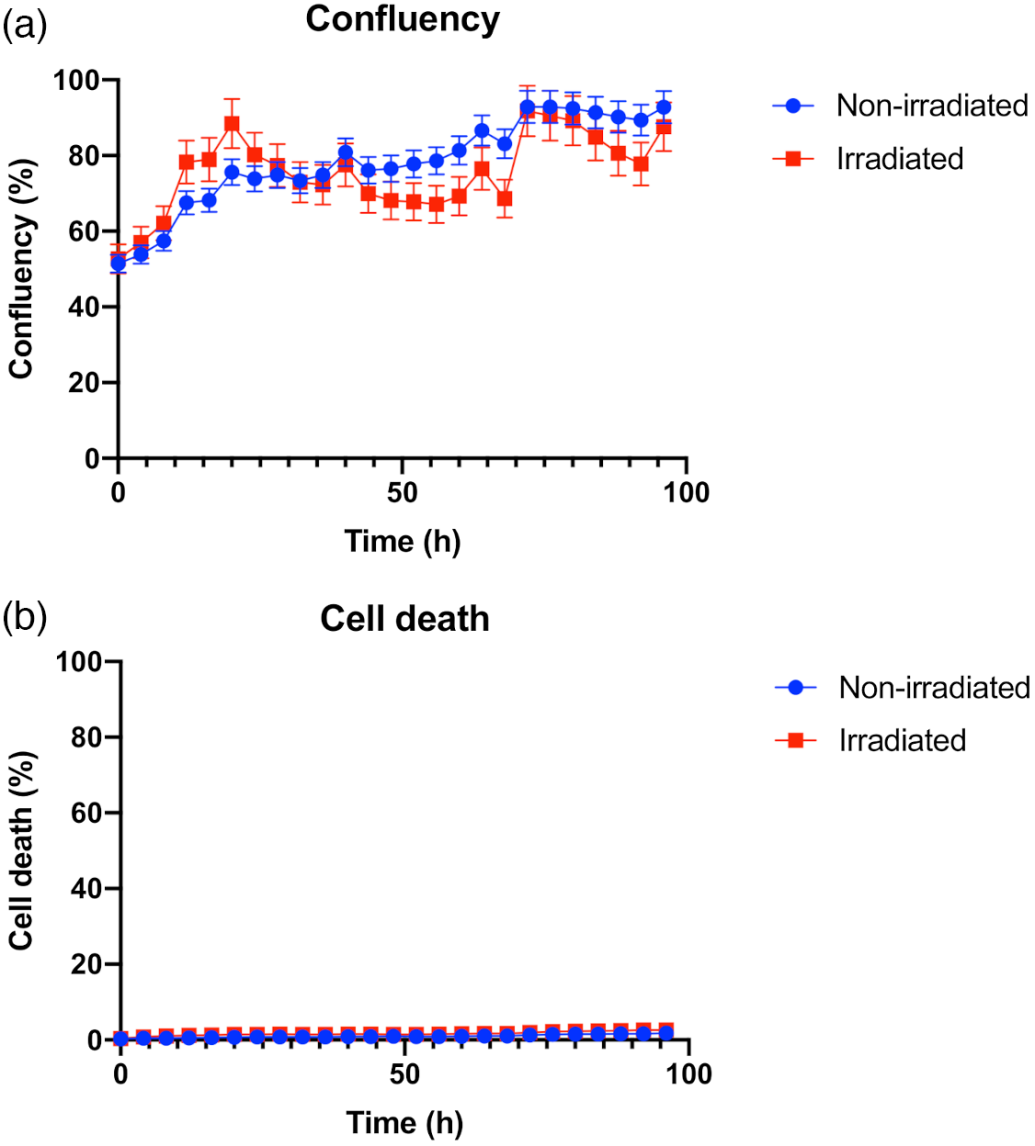
(a) Confluency (%) and (b) cell death (%) as a function of time for irradiated and control MCF7 cells. These results are the mean values of three trials with error bars (± 1 SD) and were measured using the Incucyte S3 live cell imaging system.

Cell confluency and death was measured every 4 hrs using an Incucyte S3 Live Cell Imaging system. We found that irradiation was not associated with any significant cell death up to 72 hrs after irradiation [Fig. 1(b)]. Cell confluency increased at similar rates in both control and irradiated cells up to 24 hrs but diverged between 24 and 72 hrs. While the confluency of control cells remained >75% confluency, the confluency of the irradiated population dropped <75%. At 72 hrs, the confluency of control cells was close to 100% for control but only neared 90% for irradiated cells. Consistent with previous studies,^32^ these changes are likely associated with delays in proliferation and growth between the control and irradiated cells.

### 3.2 Radiation-Induced Lipid Changes in MCF7 Cells

**Fig. 2:**
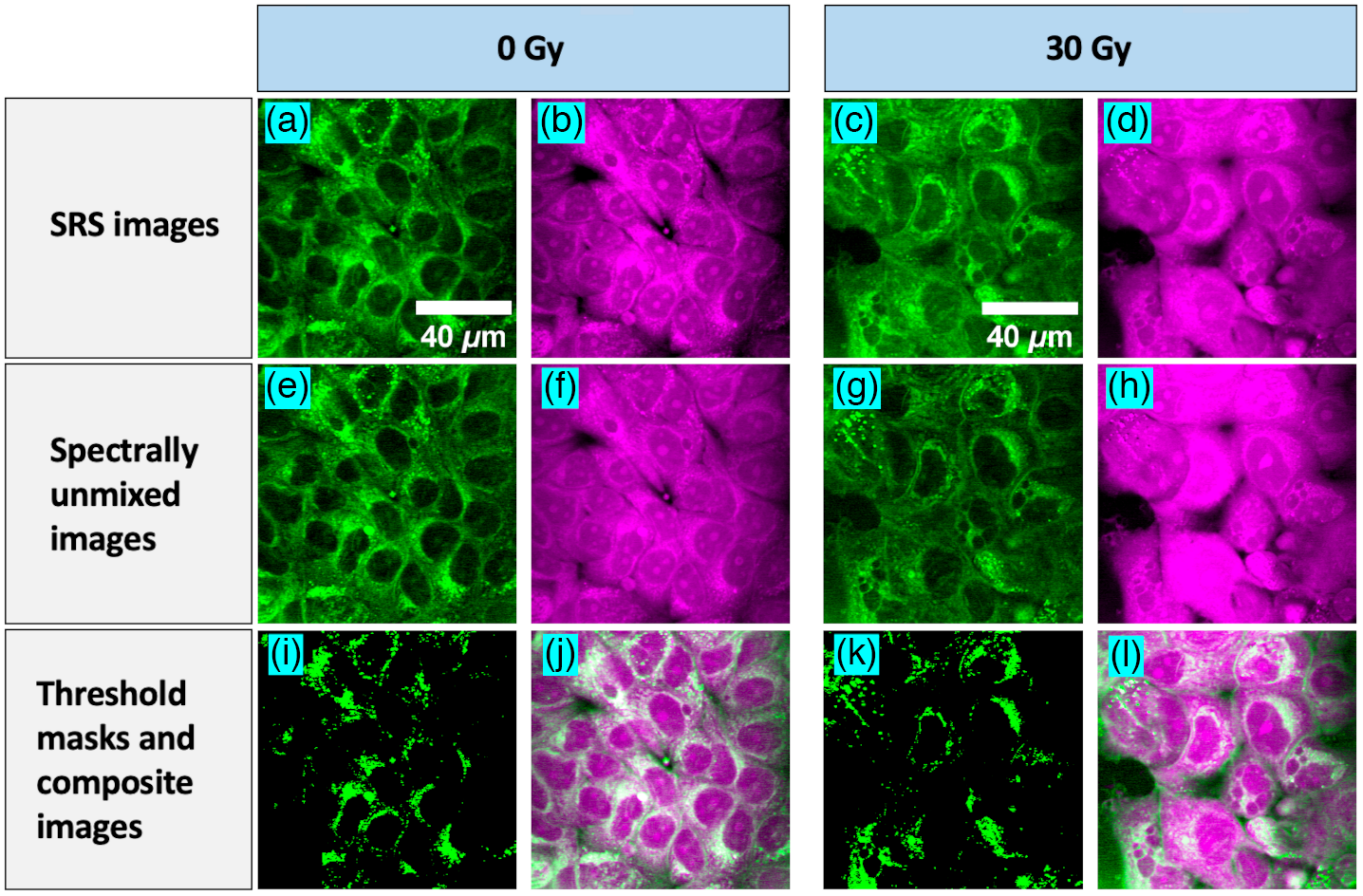
Representative SRS images of MCF7 cells taken at $2850\;{\mathrm{cm}}^{-1}$ (a,c) and $2926\;{\mathrm{cm}}^{-1}$ (b,d) for unirradiated (a,b) and irradiated (c,d). The corresponding spectrally unmixed lipid-rich (e,g) and protein-rich (f,h) images after applying MCR-ALS. The threshold masks (i,k) from the above lipid images and composite lipid-protein images (j,l).

To determine the changes in lipid metabolism, we imaged both unirradiated (0 Gy) and irradiated (30 Gy) MCF7 cells at the $2850\;{\mathrm{cm}}^{-1}$ (${\mathrm{CH}}_{2}$) band with contributions predominantly from lipids, and at the $2926\;{\mathrm{cm}}^{-1}$ (${\mathrm{CH}}_{2}$, ${\mathrm{CH}}_{3}$) band with overlapping contributions from proteins and lipids. Representative SRS images of unirradiated (0 Gy) and irradiated (30 Gy) MCF7 cells at 2850 and $2926\;{\mathrm{cm}}^{-1}$ and 72 hrs post exposure are shown in Figs. 2(a)–2(d). Figures 2(e)–2(h) shows the resulting spectrally unmixed images with the SRS images of lipid concentration showing darker nuclei, as expected. The threshold masks for the lipid images are shown in Figs. 2(i) and 2(k), and the composite lipid-protein images after spectral unmixing and LD segmentation are shown in Figs. 2(j) and 2(l).

**Fig. 3:**
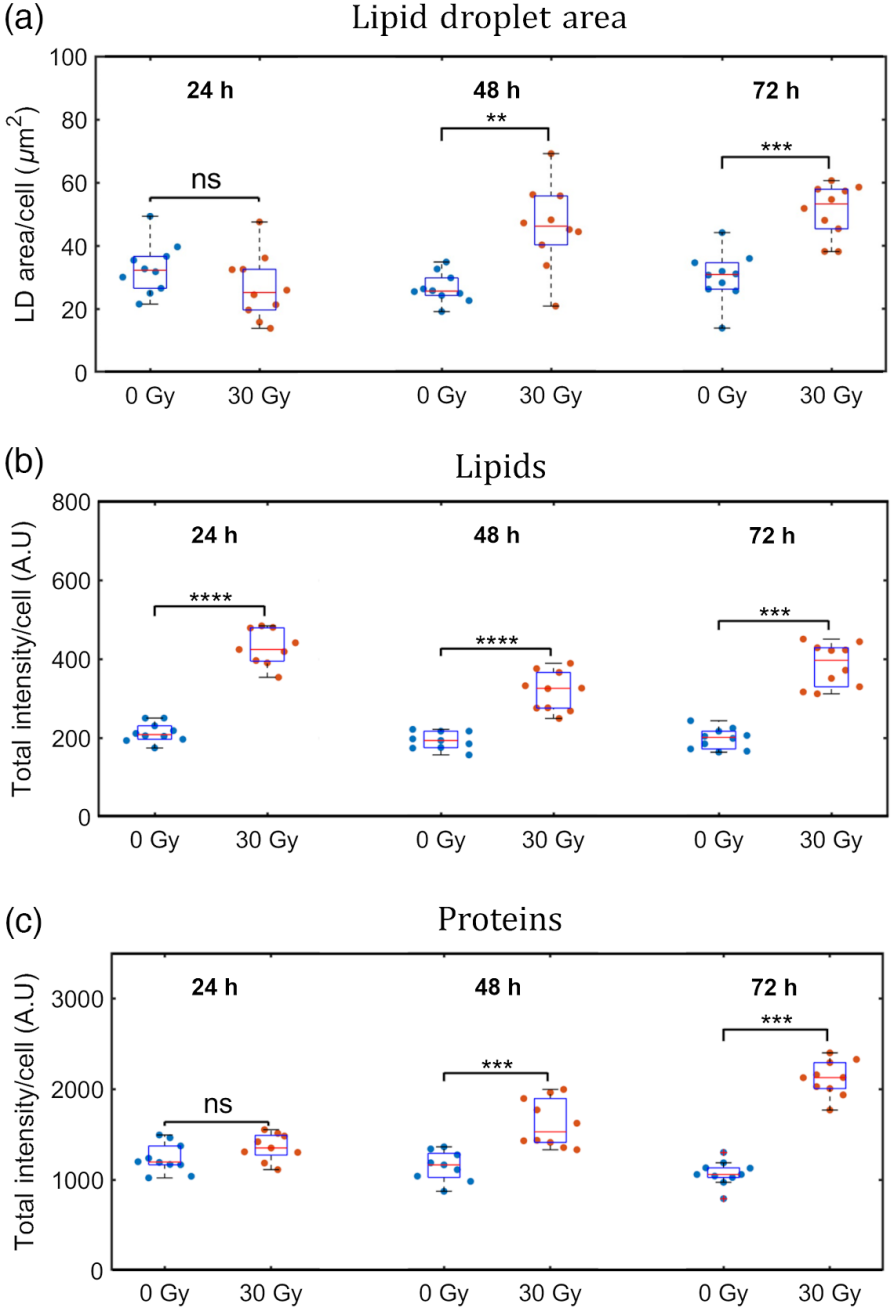
(a) Average LD area per cell, (b) total lipid intensity per cell, and (c) total protein intensity per cell over 72 hrs. LD Area/cell is the average LD area per cell for an imaged region (n=10). (ns = no significance, *p<0.05, **p<0.01, ***p<0.001, and ****p<0.0001).

To examine the time-dependent changes in the cellular lipids, we first generated segmentation masks of the LDs as described in Sec. 2.6 and evaluated the LD area per cell at the multiple time points. Figure 3 shows increasing LD area per cell with time, with a greater mean LD area per cell for irradiated than control, with this difference being most significant (p<0.001) at 72 hrs for all three trials. The total lipid and protein intensities per cell were also measured and found to show a significant increase after 48 and 72 hrs in the 30 Gy-irradiated cells compared to the control, as can be seen in Fig. 3.

### 3.3 Differences in the Cellular Morphology of Control and Irradiated Cells

**Fig. 4:**
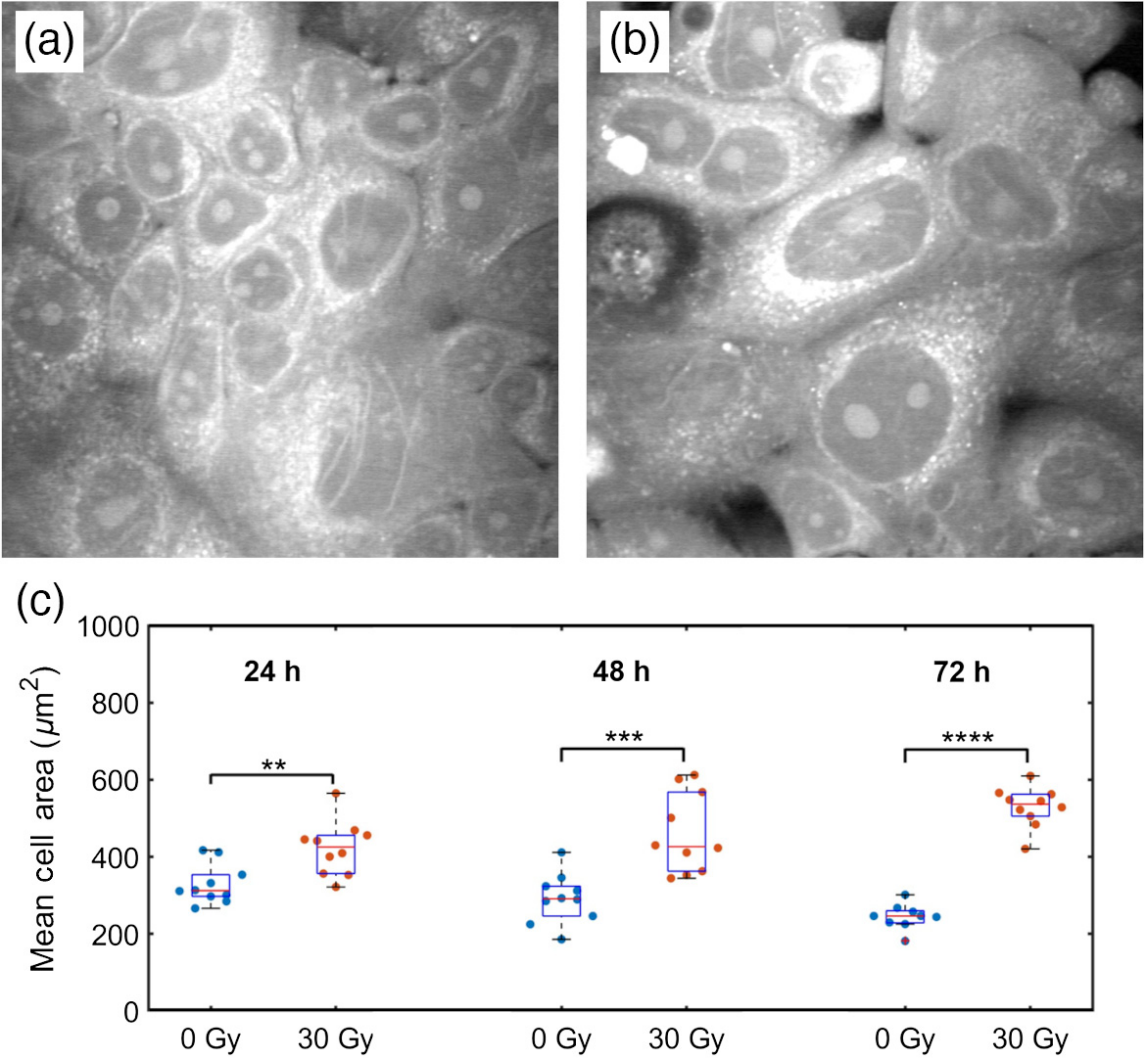
Representative cell images at $2926\;{\mathrm{cm}}^{-1}$ for (a) 0 Gy and (b) 30 Gy at 72 hrs. (c) Mean cell area at each time point where the cell area was determined from protein images (n=10). (**p<0.01, ***p<0.001, and ****p<0.0001).

To determine whether the increase in LDs correlated with cellular expansion, we next evaluated the area of the control and irradiated cells at the different time points. We observed a significant increased cell size in the irradiated cells compared to control at each time point [Fig. 4], with this difference in average size increasing with time. An example of this cellular enlargement in the irradiated MCF7 cells can be seen in the representative SRS image at $2926\;{\mathrm{cm}}^{-1}$ in Fig. 4 and is characteristic of senescent cells.^11^

**Fig. 5:**
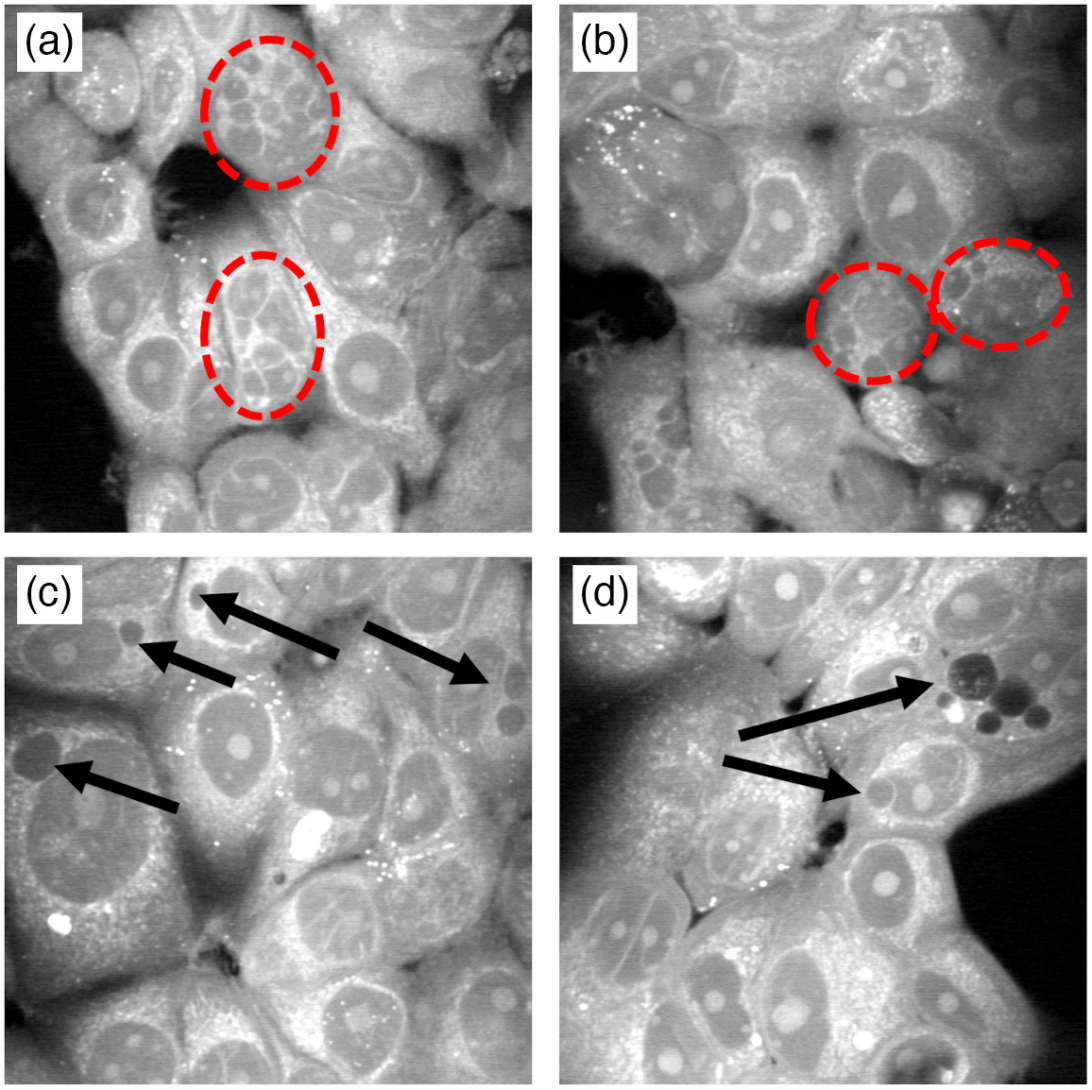
Some regions of MCF7 cells contained multi-nucleated (red circles) cells (a,b) and even more regions (c,d) contained cells containing vacuoles (arrows).

Besides the larger average cell size for irradiated cells compared to control, other morphological differences were also observed in the SRS images of the irradiated cells including increased vacuole formation in the cytoplasm and multinucleation, a sign of mitotic catastrophe [Fig. 5].

### 3.4 Lipid Peroxidation Assay

**Fig. 6:**
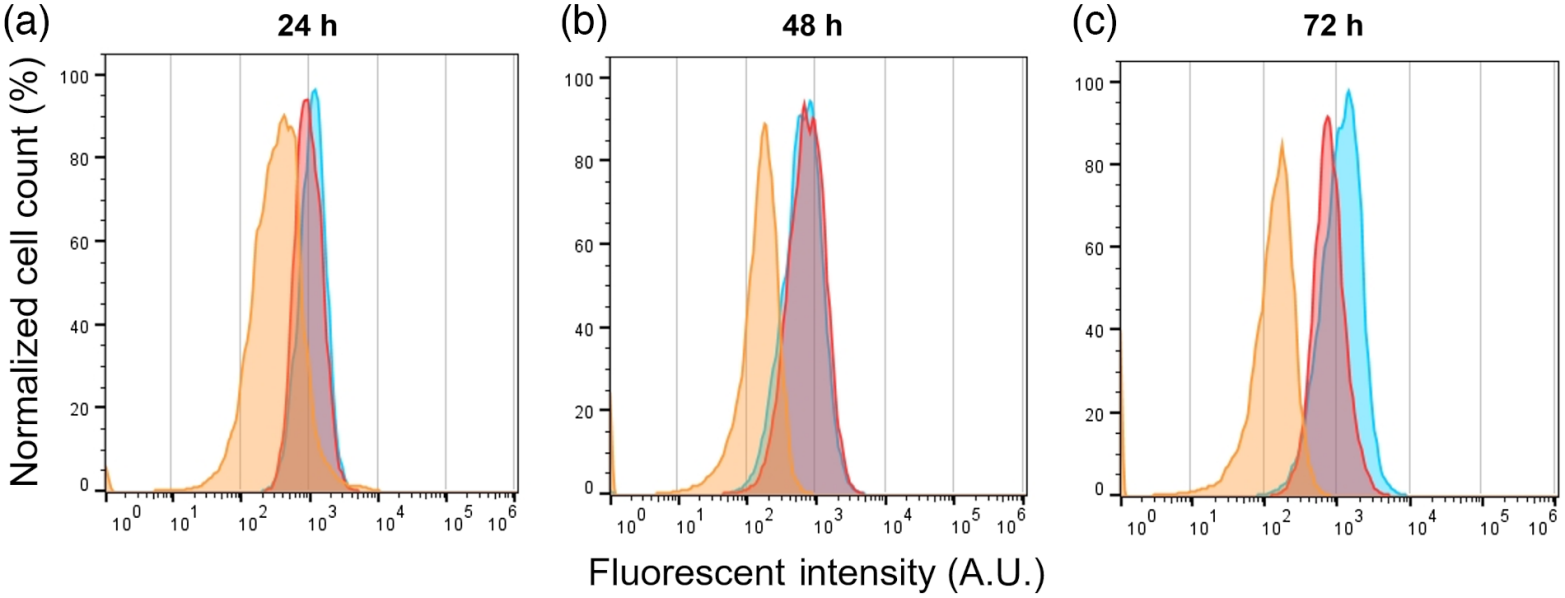
Flow cytometry data for MCF7 cells over a 3-day recovery period at (a) 24, (b) 48, and (c) 72 hrs for a single representative trial. Histograms show profiles for unstained (0 Gy;orange), control (0 Gy with BODIPY;red), and irradiated cells (30 Gy irradiated with BODIPY;blue). The Y-axis represents the normalized cell count as a percentage of the Max, and the X-axis represents the fluorescent intensity of the portion of C11-BODIPY dye that was oxidized after interaction with peroxyl radicals, emitting in the green region (490 to 510 nm) of the visible spectrum.

To determine whether the radiation exposure caused lipid peroxidation, MCF7 cells were exposed to 0 Gy or 30 Gy, stained with C11-BODIPY and analyzed by flow cytometry over a three-day recovery period. The results for a representative trial are shown in Fig. 6 where the colored histograms refer to three populations of MCF7 cells as follows: the 0 Gy is the unirradiated control group stained with BODIPY; the 30 Gy is the irradiated group stained with BODIPY, and the control group is neither irradiated nor stained with BODIPY to establish a baseline relative to the stained samples. As seen in Figs. 6(a)–6(c), a significant increase of ∼53% in the relative MFI for this trial is seen for the 30 Gy irradiated MCF7 group after 72 hrs compared to the two control groups suggesting an increase in the lipid peroxidation in irradiated MCF7 cells after 72 hrs. The average relative increase in MFI over all three trials was 67%.

## 4 Discussion

Human cancer cell lines have varying levels of intrinsic radiosensitivity.^13^^,^^33^ Here we wanted to evaluate the effects of ionizing radiation on cells with limited radiosensitivity. Hence we investigated the MCF7 human breast cancer cell line since it is known to be relatively radiation resistant with the surviving fraction after 2 Gy (${\mathrm{SF}}_{2}$) value >0.6.^13^ It is important to note that the ${\mathrm{SF}}_{2}$ value determined from a long-term clonogenic survival assay, measures the potential for proliferation post-irradiation and is different from the measurements of cell viability. Consistent with the relatively radiation-resistant phenotype, our results [Fig. 1] show no difference in % cell viability in unirradiated versus irradiated cells over the 72-hr time frame. Irradiated cells did, however, show signs of reduced proliferative potential [Fig. 1(a)]. Interestingly, MCF7 cells contain a wild type p53 gene, which is responsible for G1 phase cell cycle arrest post-irradiation.^13^^,^^33^ Therefore, irradiated MCF7 cells will remain viable after irradiation but will have a reduced clonogenic potential due to cell cycle arrest.

As seen in Fig. 3, we found a significant increase in LDs in the 30 Gy-irradiated cells at 72 hrs post-radiation. Cancer cells are known to have higher levels of LDs due to metabolic reprogramming.^34^ LD accumulation has been seen in human breast, bladder, lung, neuroglioma, and prostate cancer cells in response to 6 Gy X-rays treatment.^9^ Adjemian et al. reported a similar increase in LDs in a panel of murine cancer cells irradiated at high (>20  Gy) X-rays dose and observed cellular features related to methuosis.^11^ Their accumulation has been correlated to their survival after irradiation. It is also closely connected with iron metabolism. Free iron within the cell leads to the generation of ROS through the Fenton reaction (${{\mathrm{Fe}}_{2}}^{+}+H_{2}O_{2}\to {{\mathrm{Fe}}_{3}}^{+}+•\mathrm{OH}+{\mathrm{OH}}^{-}$). The hydroxyl radical produced causes lipid peroxidation, targeting unsaturated fatty acids. Iron chelation by desferoxamine (DFO) was able to induce LD accumulation and this, in turn, conferred higher survival ability to cells after radiation treatment, as shown by clonogenic assays.^9^ It has been suggested by Tirinato et al. that LDs could serve as an antioxidant system, being able to buffer the excess of ROS commonly found in cancer cells and prevent damage to other cellular components, such as proteins and nucleic acids. Earlier work using Raman spectroscopy found increased levels of proteins in MCF7 cells over 72-hr post irradiation^13^ that were attributed to the cellular radioadaptive response. Consistent with previous studies, we found an overall increase in the lipids and protein intensity per cell [Fig. 3] in the 30 Gy irradiated cells compared to the control cells over 72 hrs post-irradiation. Interestingly, our results of Figs. 3(b) and 4(c) indicate that the total lipid intensity per cell and the mean cell area, respectively, show significant increase at 24 hrs in irradiated cells compared to the control. On the other hand, the LD area per cell [Fig. 3(a)] and the overall protein intensity per cell [Fig. 3(c)] show comparable response in irradiated and control cells at 24 hrs. Hence, we hypothesize that lipid accumulation and endoplasmic reticulum (ER) expansion involving hypertrophy of ER, Golgi, and possibly mitochondria membranes, occur concurrently at 24 hrs post-irradiation. LD formation is induced by ER stress to maintain lipid, protein, and calcium homeostasis.^35^ In particular, hypertrophy of the smooth ER where lipids are synthesized, occurs in the initial stages of ER stress followed by LD formation. We theorize that irradiation of cells by 30 Gy X-rays may result in initial ER stress and subsequent LD formation, as has been shown in other studies.^36^ Moreover, Fig. 3(a) suggests that the irradiated cells may already be accumulating lipids from the extracellular fluid but have not yet begun an increase in production of LDs at 24 hrs.

Methuosis is a nonapoptotic mechanism characterized by massive vacuolation, accumulation of LDs, macropinocytosis, mitochondrial dysfunctions, and eventual implosive, non-autophagic cell death.^37^ Vacuoles and LDs are generated by expansion of the ER and can be induced by iron chelators, such as DFO.^38^ Exposure of cancer cells to ionizing radiation triggers an accumulation of unsaturated fatty acids that are subject to lipid peroxidation in the presence of free iron. As a compensatory effect, the induction of the ferritin heavy chain protein may sequester iron to prevent further damage from lipid peroxides but may trigger a methuosis-like state in the cell due to iron depletion. Consistent with earlier work, our SRS imaging study revealed methuosis-like features, such as cell area expansion [Fig. 4] and vacuolization [Fig. 5] in irradiated MCF7 breast cancer cells in the later stages (after 3 days) of recovery from irradiation. We also found an increase in lipid peroxidation in MCF7 cells after 72 hrs [Fig. 6] using the lipid peroxidation sensor BODIPY C11. A key difference from true methuosis is that cell death was not observed in this study. MCF7 cells continued to proliferate, albeit slower after irradiation, while displaying methuosis-like features. This may either be a contributing factor, or as a result of, their radiation resistance. Further studies on the effects of irradiation in the presence or absence of cellular iron, as well as the effects of this on lipid peroxidation and methuosis-like states in cancer cells, are required.

The radiation dose of 30 Gy investigated here is more relevant to high dose RT modalities such as IMRT and SART than conventional, 2 to 10 Gy dose regimens. Our future SRS imaging work will investigate radiation response at these lower doses, including radiation-induced effects on LDs composition at different time points. Although the dose rate used in this study was constant at 1  Gy/min during the whole irradiation period, our methods can be extended to investigate samples irradiated at different dose rates. Moreover, the SRS imaging methodology and analysis techniques demonstrated here can be readily applied to normal cells and tissue for investigating the effect of ROS in radiation-induced normal tissue damage.^6^ Our future work will simultaneously probe changes in lipids, DNA, and proteins using multiple Raman bands for SRS imaging. The use of SRS imaging strategies utilizing stable isotope labeling and bio-orthogonal tags^18^^–^^21^ will be explored to increase the molecular specificity and sensitivity of detection of radiation-induced effects.

## 5 Conclusion

This pilot study demonstrated the potential of SRS imaging for investigating ionizing radiation-induced changes in cancer cells. The high spatial resolution imaging approach enabled quantitative visualization of the changes in lipids and morphological features in 30 Gy irradiated MCF7 cells compared to unirradiated control cells, albeit without the use of fluorescent labels. Future investigations of irradiated cancer as well as normal cells and tissue using SRS imaging have the potential to provide critical insights on radiation resistance in tumors, radiation toxicity in surrounding healthy tissue, and non-targeted bystander effects. This will aid in the path toward the development of personalized RT.

## Code, Data, and Materials Availability

Data and code will be made available upon request.

## References

[1] Baskar R. et al., “Biological response of cancer cells to radiation treatment,” Front. Mol. Biosci. 1, 24 (2014). 10.3389/fmolb.2014.00024 25988165 PMC4429645

[r2] Scaife J. E. et al., “Exploiting biological and physical determinants of radiotherapy toxicity to individualize treatment,” Br. J. Radiol. 88(1051), 20150172 (2015). BJRAAP 0007-1285 10.1259/bjr.20150172 26084351 PMC4628540

[3] Hellevik T. Martinez-Zubiaurre I., “Radiotherapy and the tumor stroma: the importance of dose and fractionation,” Front. Oncol. 4, 1 (2014). FRTOA7 0071-9676 10.3389/fonc.2014.00001 24478982 PMC3896881

[4] Wilkinson B. Hill M. A. Parsons J. L., “The cellular response to complex DNA damage induced by ionising radiation,” Int. J. Mol. Sci. 24(5), 4920 (2023).1422-0067 10.3390/ijms24054920 36902352 PMC10003081

[r5] Moon J. et al., “DNA damage and its role in cancer therapeutics,” Int. J. Mol. Sci. 24(5), 4741 (2023).1422-0067 10.3390/ijms24054741 36902170 PMC10003233

[6] Wei J. et al., “Radiation-induced normal tissue damage: oxidative stress and epigenetic mechanisms,” Oxid. Med. Cell. Longev. 2019, 3010342 (2019). 10.1155/2019/3010342 31781332 PMC6875293

[7] Yuan Z. et al., “Fatty acids metabolism: the bridge between ferroptosis and ionizing radiation,” Front. Cell Dev. Biol. 9, 675617 (2021). 10.3389/fcell.2021.675617 34249928 PMC8264768

[8] Bian X. et al., “Lipid metabolism and cancer,” J. Exp. Med. 218(1), e20201606 (2021). JEMEAV 0022-1007 10.1084/jem.20201606 33601415 PMC7754673

[9] Tirinato L. et al., “Lipid droplets and ferritin heavy chain: a devilish liaison in human cancer cell radioresistance,” Elife 10, e72943 (2021). 10.7554/eLife.72943 34499029 PMC8497056

[10] Yue S. et al., “Cholesteryl ester accumulation induced by PTEN loss and PI3K/AKT activation underlies human prostate cancer aggressiveness,” Cell Metab. 19(3), 393–406 (2014).1550-4131 10.1016/j.cmet.2014.01.019 24606897 PMC3969850

[11] Adjemian S. et al., “Ionizing radiation results in a mixture of cellular outcomes including mitotic catastrophe, senescence, methuosis, and iron-dependent cell death,” Cell Death Dis. 11(11), 1003 (2020). 10.1038/s41419-020-03209-y 33230108 PMC7684309

[12] Daemen S. et al., “Microscopy tools for the investigation of intracellular lipid storage and dynamics,” Mol. Metab. 5(3), 153–163 (2016). 10.1016/j.molmet.2015.12.005 26977387 PMC4770264

[13] Matthews Q. et al., “Biochemical signatures of in vitro radiation response in human lung, breast and prostate tumour cells observed with Raman spectroscopy,” Phys. Med. Biol. 56(21), 6839 (2011). PHMBA7 0031-9155 10.1088/0031-9155/56/21/006 21971286

[r14] Harder S. J. et al., “Raman spectroscopy identifies radiation response in human non-small cell lung cancer xenografts,” Sci. Rep. 6, 21006 (2016). SRCEC3 2045-2322 10.1038/srep21006 26883914 PMC4756358

[15] Paidi S. K. et al., “Label-Free Raman spectroscopy reveals signatures of radiation resistance in the tumor microenvironmentdecoding radiation resistance with Raman spectroscopy,” Cancer Res. 79(8), 2054–2064 (2019). CNREA8 0008-5472 10.1158/0008-5472.CAN-18-2732 30819665 PMC6467810

[16] Roman M. et al., “Exploring subcellular responses of prostate cancer cells to X-ray exposure by Raman mapping,” Sci. Rep. 9, 8715 (2019). SRCEC3 2045-2322 10.1038/s41598-019-45179-y 31213635 PMC6581960

[r17] Roman M. et al., “Lipid droplets in prostate cancer cells and effect of irradiation studied by Raman microspectroscopy,” Biochim. Biophys. Acta BBA-Mol. Cell Biol. Lipids 1865(9), 158753 (2020). LPDSAP 0024-4201 10.1016/j.bbalip.2020.158753 32504818

[18] Xu J. et al., “Unveiling cancer metabolism through spontaneous and coherent Raman spectroscopy and stable isotope probing,” Cancers 13(7), 1718 (2021). 10.3390/cancers13071718 33916413 PMC8038603

[r19] Hu F. Shi L. Min W., “Biological imaging of chemical bonds by stimulated Raman scattering microscopy,” Nat. Methods 16(9), 830–842 (2019).1548-7091 10.1038/s41592-019-0538-0 31471618

[r20] Freudiger C. W. et al., “Label-free biomedical imaging with high sensitivity by stimulated Raman scattering microscopy,” Science 322(5909), 1857–1861 (2008). SCIEAS 0036-8075 10.1126/science.1165758 19095943 PMC3576036

[21] Hill A. H. Fu D., “Cellular imaging using stimulated Raman scattering microscopy,” Anal. Chem. 91(15), 9333–9342 (2019). ANCHAM 0003-2700 10.1021/acs.analchem.9b02095 31287649

[22] Li J. et al., “Lipid desaturation is a metabolic marker and therapeutic target of ovarian cancer stem cells,” Cell Stem Cell 20(3), 303–314.e5 (2017). 10.1016/j.stem.2016.11.004 28041894 PMC5337165

[23] Hislop E. W. et al., “Label-free imaging of lipid droplets in prostate cells using stimulated Raman scattering microscopy and multivariate analysis,” Anal. Chem. 94(25), 8899–8908 (2022). ANCHAM 0003-2700 10.1021/acs.analchem.2c00236 35699644 PMC9244870

[24] Tipping W. J. et al., “Stimulated Raman scattering microscopy with spectral phasor analysis: applications in assessing drug–cell interactions,” Chem. Sci. 13(12), 3468–3476 (2022).1478-6524 10.1039/D1SC06976D 35432863 PMC8943890

[25] Allen C. H. et al., “Imaging radiobiological response of breast cancer cells in vitro using stimulated Raman scattering,” Proc. SPIE 12392, 1239204 (2023). PSISDG 0277-786X 10.1117/12.2650138

[26] Allen C. H. et al., “Coherent anti-Stokes Raman scattering imaging using silicon photomultipliers,” Opt. Lett. 45(8), 2299–2302 (2020). OPLEDP 0146-9592 10.1364/OL.390050 32287218

[27] Gagnon J. R. et al., “Spectral focusing-based stimulated Raman scattering microscopy using compact glass blocks for adjustable dispersion,” Biomed. Opt. Express 14, 2510–2522 (2023). BOEICL 2156-7085 10.1364/BOE.486753 37342685 PMC10278629

[28] Pologruto T. A. Sabatini B. L. Svoboda K., “ScanImage: flexible software for operating laser scanning microscopes,” Biomed. Eng. Online 2, 13 (2003). 10.1186/1475-925X-2-13 12801419 PMC161784

[29] Galli R. et al., “Intrinsic indicator of photodamage during label-free multiphoton microscopy of cells and tissues,” PloS One 9(10), e110295 (2014). POLNCL 1932-6203 10.1371/journal.pone.0110295 25343251 PMC4208781

[30] Figueroa B. et al., “Real-time microscale temperature imaging by stimulated Raman scattering,” J. Phys. Chem. Lett. 11(17), 7083–7089 (2020). JPCLCD 1948-7185 10.1021/acs.jpclett.0c02029 32786960

[31] Jaumot J. de Juan A. Tauler R., “MCR-ALS GUI 2.0: new features and applications,” Chemom. Intell. Lab. Syst. 140, 1–12 (2015). 10.1016/j.chemolab.2014.10.003

[32] Villalobos M., “Radiosensitivity of human breast cancer cell lines of different hormonal responsiveness. Modulatory effects of oestradiol,” Int. J. Radiat. Biol. 70(2), 161–169 (1996). IJRBE7 0955-3002 10.1080/095530096145157 8794845

[33] McIlwrath A. J. et al., “Cell cycle arrests and radiosensitivity of human tumor cell lines: dependence on wild-type p53 for radiosensitivity,” Cancer Res. 54(14), 3718–3722 (1994). CNREA8 0008-5472 8033090

[34] Ward P. S. Thompson C. B., “Metabolic reprogramming: a cancer hallmark even Warburg did not anticipate,” Cancer Cell 21(3), 297–308 (2012). 10.1016/j.ccr.2012.02.014 22439925 PMC3311998

[35] Jarc E. Petan T., “Focus: organelles: lipid droplets and the management of cellular stress,” Yale J. Biol. Med. 92(3), 435 (2019). 31543707 PMC6747940

[36] Kim W. et al., “Cellular stress responses in radiotherapy,” Cells 8(9), 1105 (2019). 10.3390/cells8091105 31540530 PMC6769573

[37] Maltese W. A. Overmeyer J. H., “Methuosis: nonapoptotic cell death associated with vacuolization of macropinosome and endosome compartments,” Am. J. Pathol. 184(6), 1630–1642 (2014). AJPAA4 0002-9440 10.1016/j.ajpath.2014.02.028 24726643 PMC4044715

[38] De Bortoli M. et al., “Lipid accumulation in human breast cancer cells injured by iron depletors,” J. Exp. Clin. Cancer Res. 37, 75 (2018). 10.1186/s13046-018-0737-z 29615075 PMC5883539

